# The impact of digital interventions on health insurance coverage for reproductive, maternal, newborn and child health services utilization in Kakamega, Kenya: a cluster randomized controlled trial

**DOI:** 10.1093/heapol/czae079

**Published:** 2024-08-24

**Authors:** Amanuel Abajobir, Richard de Groot, Caroline Wainaina, Menno Pradhan, Wendy Janssens, Estelle M Sidze

**Affiliations:** Sexual, Reproductive, Maternal, Newborn, Child, and Adolescent Health Unit, African Population and Health Research Center, APHRC Campus, Kitisuru, Nairobi 10787-00100, Kenya; Amsterdam Institute of Global Health and Development, Amsterdam, The Netherlands; Sexual, Reproductive, Maternal, Newborn, Child, and Adolescent Health Unit, African Population and Health Research Center, APHRC Campus, Kitisuru, Nairobi 10787-00100, Kenya; Amsterdam Institute of Global Health and Development, Amsterdam, The Netherlands; Vrije Universiteit, Amsterdam, The Netherlands; Universiteit van Amsterdam, The Netherlands; Amsterdam Institute of Global Health and Development, Amsterdam, The Netherlands; Vrije Universiteit, Amsterdam, The Netherlands; Sexual, Reproductive, Maternal, Newborn, Child, and Adolescent Health Unit, African Population and Health Research Center, APHRC Campus, Kitisuru, Nairobi 10787-00100, Kenya

**Keywords:** Maternal, newborn and child health, universal health coverage, health insurance, digital health insurance, Kenya

## Abstract

The National Hospital Insurance Fund (NHIF) of Kenya was upgraded to improve access to healthcare for impoverished households, expand universal health coverage, and boost the uptake of essential reproductive, maternal, newborn and child health (RMNCH) services. However, premiums may be unaffordable for the poorest households. The Innovative Partnership for Universal Sustainable Healthcare (*i*-PUSH) programme targets low-income women and their households to improve their access to and utilization of quality healthcare, including RMNCH services, by providing subsidized, mobile phone-based NHIF coverage in combination with enhanced, digital training of community health volunteers and upgrading of health facilities. This study evaluated whether expanded NHIF coverage increased the accessibility and utilization of quality basic RMNCH services in areas where *i*-PUSH was implemented using a longitudinal cluster randomized controlled trial in Kakamega, Kenya. A total of 24 pair-matched villages were randomly assigned either to the treatment or the control group. Within each village, 10 eligible households (i.e. with a woman aged 15–49 years who was either pregnant or with a child <4 years old) were randomly selected. The study applied a difference-in-difference methodology based on a pooled cross-sectional analysis of baseline, midline and endline data, with robustness checks based on balanced panels and Analysis of Covariance methods. The analysis sample included 346 women, of whom 248 had had a live birth in the 3 years prior to any of the surveys, and 424 children aged 0–59 months. Improved NHIF coverage did not have a statistically significant impact on any of the RMNCH outcome indicators at midline nor endline. Uptake of RMNCH services, however, improved substantially in both control and treatment areas at endline compared to baseline. For instance, significant increases were observed in the number of antenatal care visits from baseline to midline (mean = 2.62–2.92, *P* < 0.01) and delivery with a skilled birth attendant from baseline to midline (mean = 0.91–0.97, *P* < 0.01). Expanded NHIF coverage, providing enhanced access to RMNCH services of unlimited duration at both public and private facilities, did not result in an increased uptake of care, in a context where access to basic public RMNCH services was already widespread. However, the positive overall trend in RMNCH utilization indicators, in a period of constrained access due to the COVID-19 pandemic, suggests that the other components of the *i*-PUSH programme may have been beneficial. Further research is needed to better understand how the provision of insurance, enhanced community health volunteer training and improved healthcare quality interact to ensure pregnant women and young children can make full use of the continuum of care.

Key messages

- The National Hospital Insurance Fund (NHIF) in Kenya was upgraded to improve healthcare coverage for impoverished households and expand universal health coverage for reproductive, maternal, newborn and child health (RMNCH) services.
- The Innovative Partnership for Universal Sustainable Healthcare (*i*-PUSH) programme, a mobile phone-based subsidized health insurance, aimed to improve access to and utilization of quality RMNCH services, but expanded NHIF coverage did not have a statistically significant impact on the RMNCH outcomes.
- Despite the lack of significant impact on RMNCH services through NHIF coverage, the overall trend showed a substantial improvement in the uptake of RMNCH services in both control and treatment areas.
- The study suggests that while expanded NHIF coverage alone may not increase RMNCH service uptake, the other components of *i*-PUSH may have played a role in the positive overall trend observed.

## Introduction

Despite substantial progress in the past decades, maternal, newborn and under-5 child morbidity and mortality remain high in Sub-Saharan Africa (SSA) (Onambele *et al*., 2023). SSA has some of the highest mortality rates globally, with ∼533 maternal deaths per 100 000 live births (WHO, UNICEF, UNFPA, World Bank Group, and the United Nations Population Division, 2019) and ∼76 under-5 deaths per 1000 live births, leading to an estimated 2.8 million children under the age of 5 years dying each year (UN IGME, 2021). Although Kenya performs better than the regional average, mortality rates remain persistently high, with 342 maternal deaths per 100 000 live births, 52 under-5 years deaths per 1000 live births, and 39 infant deaths per 1000 live births (Kenya National Bureau of Statistics (KNBS), 2022). In Western Kenya, infant mortality rates are significantly higher than the national average (Kenya National Bureau of Statistics (KNBS), 2022).

The use of essential reproductive, maternal, newborn and child healthcare (RMNCH) services, such as antenatal care (ANC), care by a skilled birth attendant (SBA), emergency obstetric care and immediate postnatal care (PNC) for every newborn baby, are key intervention strategies to reduce mortality rates (UNICEF, 2015). Despite increased access to healthcare in SSA, service quality and content still have gaps (Sife *et al*., 2017). The scarcity of human resources also hinders efforts to enhance access to care (Kruk *et al*., 2018). Only 58% of Kenyan women received ≥ 4 ANC visits, 62% had SBA and 53% received PNC for their most recent live birth (Kenya National Bureau of Statistics (KNBS), 2022).

To enhance RMNCH outcomes, it is crucial to increase access to and utilization of healthcare during pregnancy, childbirth, postpartum and early childhood. Empowering individuals, families and communities to practice self-care, preventive care and care-seeking has proven to be effective (WHO, 2011). However, this can be challenging for impoverished populations who face obstacles such as unaffordable healthcare costs, lack of information, and cultural attitudes or beliefs that hinder access to healthcare (Ngabo *et al*., 2012). Ensuring a continuum of care for mothers and newborns requires better access to care and financial protection for these vulnerable populations (Kruk *et al*., 2018).

Mobile health (mHealth) interventions can bridge the gaps in access to high-quality RMNCH services (Fadlallah *et al*., 2018; Amref Health Africa, 2020). These interventions provide women of reproductive age, including expectant mothers, with vital information and services via mobile phones, leading to increased deliveries by trained personnel, improved access to expert advice through e-learning platforms for health workers and improved referrals for complications (Spaan *et al*., 2012; Sife *et al*., 2017). In addition, mHealth platforms offer e-reminders for ANC, PNC and vaccinations, as well as improved record-keeping through digital information systems (Hemming *et al*., 2011; Fadlallah *et al*., 2018; Onambele *et al*., 2023).

Innovative mHealth approaches can be particularly effective in SSA, which has been at the forefront of the mobile revolution (Aker and Isaac, 2010; Aranda-Jan *et al*., 2014). These programmes can reach low-income households in remote areas, but they also face challenges, including operational costs, limited knowledge, inadequate infrastructure and regulatory issues. Moreover, many RMNCH mHealth programmes lack integration across the continuum of care (Kannisto *et al*., 2014; Watterson *et al*., 2015). More evidence is hence needed for effective policy formulation and implementation.

Health insurance is critical for enhancing healthcare access and quality, including for RMNCH (Crawford *et al*., 2014). It protects against financial setbacks, reduces health-related out-of-pocket expenditure and increases healthcare utilization (Alpay *et al*., 2010; Wakadha *et al*., 2013; Lara *et al*., 2017). However, high premiums can prevent the most vulnerable households—that are most likely to be benefit from the scheme—from affording insurance, leaving them uninsured. Each year, ∼100 million people are pushed below the poverty line due to uninsured health shocks (Wagstaff *et al*., 2014; WHO, 2021).

Amidst these challenges, Amref Health Africa and PharmAccess Foundation developed the Innovative Partnership for Universal Sustainable Healthcare (*i*-PUSH), a multi-pronged initiative using digital solutions both on the demand and the supply side, to enhance access to healthcare and provide financial protection for low-income households (Abajobir *et al*., 2021). It provided fully subsidized, mobile phone-based National Hospital Insurance Fund (NHIF) coverage to low-income women of reproductive age and their households; it upgraded the quality of selected health facilities, including the provision of a health information system on a mobile platform; and it enhanced the training of community health volunteers (CHVs) through a mobile phone app. Streamlined digital enrolment processes mitigated logistical barriers and time constraints associated with traditional enrolment procedures.

This article investigates the impact of the *i*-PUSH programme targeting low-income women—who were either pregnant or with a child below 4 years of age—and their households in rural Western Kenya on maternal and newborn health outcomes. Using a cluster-randomized controlled trial (RCT) design, the study evaluates the impact of the fully subsidized, mobile phone-based insurance component on access to and use of RMNCH services and outcomes, combining real-world implementation with a rigorous evaluation of outcomes.

## Methods

### Context of health insurance in Kenya

Kenya’s government is striving for universal health coverage for all citizens, particularly those in vulnerable situations, through policy reforms and strategic initiatives. The Linda Mama programme was introduced in 2013 to provided free maternal health services by eliminating user fees at government primary healthcare facilities (Orangi *et al*., 2021). Since 2017, the NHIF abolished user fees for maternity services as well in all (primary, secondary and tertiary) public health facilities contracted by NHIF. NHIF coverage includes the same benefit package of antenatal, delivery and postnatal services as Linda Mama, but without time limits, whereas Linda Mama only covers the first 12 months after the pregnancy was recorded. In addition, NHIF enables women to seek health services from other registered NHIF contracted providers, including private providers. This offers benefits when women are unable to get the essential services such as ultrasound and medicines at a public health facility (Comfort *et al*., 2013). Thus, the expectation is that NHIF coverage will further enhance women’s access to ‘unlimited’ and better quality (primary, secondary and tertiary public and private) maternal care as compared to the Linda Mama programme. However, enrolment in NHIF remains limited, especially among the poorer, non-indigent households and informal sector workers, who do not qualify for government-subsidized schemes, so that premium payments are an obstacle while enrolment is voluntary. Logistical barriers and paperwork (e.g. the need for birth certificates or ID cards) are other obstacles to enrolment.

### Description of the i-PUSH programme and its context

The *i*-PUSH intervention provides free NHIF ‘SupaCover’, the standard comprehensive NHIF health insurance package, including RMNCH care, to low-income populations, particularly women of reproductive age and their families in Kakamega and Nairobi Counties. First, it offers fully subsidized, digital insurance, relieving financial constraints. The insurance is registered on a woman’s own sim card, to empower women and provide them with more direct decision-making control over healthcare utilization, as they do not need to request the insurance card from their husbands. *i*-PUSH enrolment officers come to a woman’s home or workplace and provide support in digitally uploading via the woman’s phone all required documentation such as photocopies of ID-cards and birth certificates, in order to relieve logistical and administrative constraints. This component is hence expected to further increase uptake of NHIF health insurance and to subsequently facilitate unlimited access to RMNCH services (at NHIF providers of all levels, including private facilities). A second key component consists of PharmAccess’ SafeCare quality enhancement programme for selected NHIF-affiliated facilities, which improves quality of care for RMNHC as well as non-RMNHC services (Johnson *et al*., 2016), while linking them to a digital health platform (called M-TIBA) that connects patients, healthcare providers and the NHIF on a real-time basis to stimulate data sharing and enhance efficiency, transparency and accountability in the system. The third component focuses on CHVs, who are supported with enhanced RMNHC skills training through the mobile phone-based LEAP training tool. Appendix 1 provides further details of the intervention using the TIDIER framework (Hoffmann *et al*., 2014) (Appendix 1; supplementary file 1, see supplementary online material).

The intervention thus addresses various household constraints, health facility quality and CHV skills, which is expected to increase RMNCH service utilization and provision, ultimately aiming to improve RMNCH outcomes. Our cluster-RCT focuses solely on the impact of providing fully subsidized enrolment into NHIF insurance for the duration of a full year, which was offered with logistical and administrative support to a random selection of women and their newborn children in the study communities. The quality upgrade of facilities and the impact of the CHV training tool are not part of this evaluation because all study communities had access to the upgraded facilities, and the CHVs in both treatment and control communities were trained on LEAP.

### Study site and study period

Our study concentrates on Kakamega County. The *i*-PUSH programme has been implemented in multiple sub-counties of Kakamega County since 2017. The cluster-RCT was conducted in Khwisero sub-county, one of the sub-counties into which *i*-PUSH expanded in 2020. Four healthcare providers in Khwisero sub-county had attained NHIF level 4 at baseline, providing both out- and in-patient care, and were hence eligible for NHIF-empanelment and to be included in the study. These four health facilities (one public and three private) were invited by PharmAccess and Amref to participate in the SafeCare Quality Improvement Programme in the autumn of 2019. The *i*-PUSH programme introduced subsidized health insurance to all selected households in treatment communities (but not control communities) from June to October 2020, while CHVs in the entire study area (i.e. both treatment and control communities) received LEAP training from October 2020 onwards. Data were collected at baseline (October–November 2019), midline (December 2020) and endline (June–July 2021). After the outbreak of COVID-19 in March 2020, in person interviews were replaced with phone interviews.

### Study design

A matched-pair longitudinal cluster-RCT was conducted, ensuring robustness through three steps. First, four health facilities were selected, as described above. The sub-county government provided a list of all villages in the catchment area of the four health facilities (*n* = 239), of which 24 were randomly selected for inclusion in the study (6 per health facility). Second, after the baseline survey, the 6 villages per facility were matched in pairs as similar as possible, based on relevant community characteristics and baseline household outcomes, using the Euclidean distance method (see Appendix 2 for more details on the study design), and within each pair randomly assigned to either the treatment or the control arm. This process aimed to create balanced treatment and control groups, considering factors like village population size, public infrastructure, literacy rates and health indicators. Randomization of villages was done in a public ceremony, with village leaders and stakeholders present, assigning villages to treatment or control using a coin flip. Third, based on a complete household listing, in each village 10 eligible households were selected to be included in the study, as described below, to reach a total sample size of 240 households.

Eligible households in the treatment villages received access to free insurance after the baseline survey. Eligible households in the control villages received free insurance after endline. Households in both groups had access to improved quality of care at the upgraded facilities and were served by better-trained CHVs, but only treatment households could access care at highly reduced costs. This design allows us to isolate the demand-side effect of insurance from the impact of the other *i*-PUSH components, while controlling for supply-side improvements in quality of care.

For programmatic reasons, two of the four selected facilities, both private, were later dropped from *i*-PUSH and did not participate in the SafeCare quality improvement plan. The treatment and control clusters selected from their catchment areas were kept in the study sample as they were all located within easy reach of one of the remaining upgraded facilities, one public and one private. Given the intervention’s focus on communities and health facilities, this cluster-RCT method ensures that the intervention is implemented at the community level, mirroring real-world scenarios, yet still enabling a rigorous evaluation of the impact of subsidized health insurance on individual health outcomes. The design does not allow for an evaluation of enhanced quality of care because all households in the study area, regardless of treatment status, could benefit from the well-trained CHVs and upgraded facilities. Further information can be found in the published protocol (Abajobir *et al*., 2021) and in two trial registries (AEA Registry [AEARCTR-0006089] and ClinicalTrials.gov [NCT04068571]).

### Study participants

On average, a village consisted of ∼100 households, each served by a separate CHV and within reach of one of the upgraded facilities. The area manager of PharmAccess Foundation together with the CHVs provided the full list of households and relevant information for each study village (both treatment and control) to the research team. Eligible households were identified based on their demographic characteristics and pregnancy status. Eligible households were those with at least one woman of reproductive age (18–49 years) who either had a child under the age of 4 years living with her at baseline or was pregnant at that time. Additionally, teenage girls aged 15–19 years who had a child under 4 years old or were pregnant (‘emancipated minor’) were also considered eligible. All members of sampled eligible households provided data for the study.

The original design was to have an equal number of pregnant women and families with children under 4 years old, but this was not possible due to the low number of pregnant women. Therefore, all pregnant women were sampled, and additional households with young children were randomly selected until a cluster size of 10 households per village was achieved. The total planned sample size was 240 households (120 households in the treatment group and 120 households in the control group). A random selection method was used to select households with young children into the study from the list of all eligible households with children below 4 years of age, using a spreadsheet and a randomly allocated number. Additional eligible households with children below 4 years were over-sampled to account for potential dropouts. Some households included multiple eligible women, who were all included in the data collection to capture a comprehensive picture of the household’s healthcare experiences and to avoid omitting eligible women, which meant that there were more women than households in the study.

### Outcome variables

The primary outcome of the trial was individual healthcare utilization, quantified by the total number of visits per individual to formal healthcare providers for treatment of any illness or injury during the study period. Programme impact on healthcare utilization and financial protection is reported elsewhere (de Groot *et al*., 2023). In this study, we evaluated the impact of *i*-PUSH on secondary outcomes, including RMNCH healthcare utilization, family planning and preventive healthcare services for children.

Women were asked about their ‘RMNCH service utilization’ for their most recent live birth during the pregnancy, childbirth and postpartum period. The data collected were self-reported and included: any ANC visit (no/yes) and if yes, its frequency (total number of ANC visits—only once, 2–3 times, ≥ 4 times), whether an SBA was present during delivery (no/yes), whether immediate PNC was received within 2 days of delivery (no/yes), as well as PNC received after returning home (no/yes). The perceived quality of ANC and delivery services was also assessed using a five-point scale ranging from very poor to very good, to assess whether perceptions of the quality of received care changed, as this might be an unintended negative consequence of becoming insured (Duku *et al*., 2018). Binary indicators were created for observations that rated the quality ‘good’ or ‘very good’. Additionally, data on the utilization of preventive services during pregnancy were collected, including tetanus toxoid injection (no/yes), iron supplements (no/yes), folic acid (no/yes) and bednets (no/yes).

The outcome variables related to reproductive health included use of modern ‘family planning’ commodities and if so, which type (no/yes; short-term/long-term) and unmet need for family planning commodities for married/partnered women who wanted to avoid or delay pregnancy but were not using any contraceptive method (no/yes) (Bradley *et al*., 2012).

RMNCH data for all children below 5 years of age at the time of each survey focused on the utilization of ‘preventive services’. These data were collected from mothers, other caregivers or knowledgeable household members. The data included vaccination uptake, excluding children under 9 months old at each survey because they could not have completed their full vaccination cycle yet. For the vaccination outcomes, a summary binary variable was created to indicate whether the child was fully immunized, based on its uptake of all basic vaccines ((Bacillus Calmette-Guérin (BCG), 4 doses of polio, 3 doses of Diphtheria, Pertussis, and Tetanus (DPT), and measles)), and a measure of the total number of vaccines received, ranging from zero to nine. The data also measured the use of bednets to prevent malaria (no/yes).

### Covariates

Covariates included the woman’s age, the woman’s literacy and a household wealth index. The wealth index was determined by analysing household ownership of various assets, including water access, sanitation facilities, domestic assets, sources of energy, means of transport and space availability, as well as animal/land ownership. The index was created using principal component analysis, and households were ranked as belonging to the poor, middle or rich tercile based on the index.

### Power calculations

**Figure 1.**
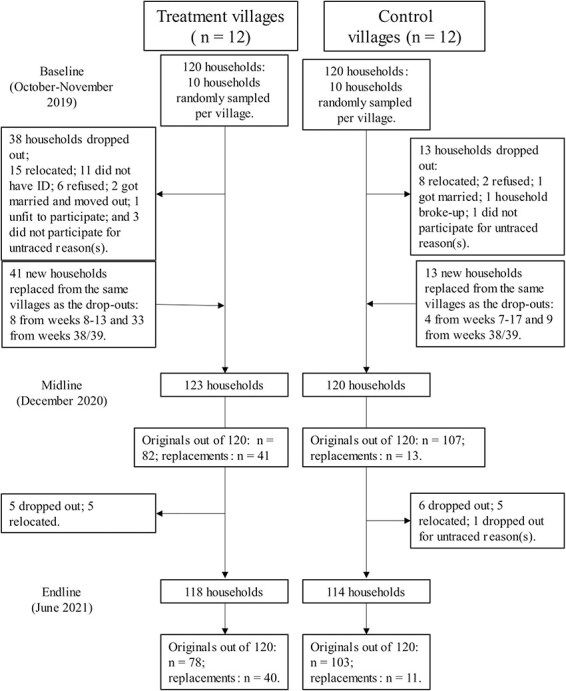
Flow diagram based on CONSORT guidelines for cluster-RCT studies

Sample-size calculations for the overall RCT were based on Hemming’s method (Hemming *et al*., 2011), with healthcare utilization at any formal facility for any type of illness or injury as the primary outcome (de Groot *et al*., 2023). With a sample of 12 clusters per treatment arm and 10 households per cluster, we estimated to be able to detect an effect size of at least a 0.4 increase in the average number of healthcare visits per individual per year (from an estimated baseline average of 5.6 visits per year for uninsured individuals, i.e. a 7% increase), based on an intra-cluster correlation of 0.014 (Geng *et al*., 2018), a 5% margin of error and 80% power. Therefore, the total sample size for the RCT was 240 households, each with at least one target woman. Additional eligible women per household would increase power. To ensure a sufficient sample size, we replaced households that dropped out of the study before the intervention began on a rolling basis with new eligible households until the programme began in June 2020. Most replacements were related to relocation after the onset of COVID-19. In total, 40 and 11 women (and their respective households) from the treatment and control groups, respectively, were replaced because they had out-migrated, did not possess a national ID-card or refused to participate. These women and their households, as well as their replacements, had similar characteristics at baseline to those included in our analysis from the start. Although more households dropped out from the treatment group than the control group (due in part to missing ID-cards being identified for treatment but not for control households), there were no significant observed differences between drop-out in the two groups. Figure 1 describes the processes and flow involved in this study based on Consolidated Standards of Reporting Trials (CONSORT) guidelines for cluster-RCT studies, including matching, pairing and randomization, implementation, and analysis.

While the original design focused on health care utilization as primary outcome, in this paper we studied several secondary outcomes. Using the means, standard deviations and intra-cluster correlations from the baseline survey data, we conducted ex-post sample size calculations of the main secondary outcomes. The minimum detectable effect (MDE) sizes at 80% power and an alpha of 0.05 are 0.166 for the use of any modern contraceptive (from a baseline value of 0.475), 0.183 for a dummy indicator of four ANC visits or more (from a baseline of 0.736), 0.192 for immediate PNC (from a baseline of 0.785) and 0.148 for fully vaccinated children under 5 years of age (from a baseline of 0.799) (Appendix 3). These MDEs are conservative upper bounds as they do not take into account the pairwise matching of clusters at randomization and the inclusion of covariates, both of which increase power and precision (Imai *et al*., 2009; Benkeser *et al*., 2021), nor the additional eligible women per household.

### Data collection and quality control

Data were collected using baseline, midline and endline survey questionnaires, which included modules on individual and household socio-demographics and economic characteristics, as well as RMNCH services utilization. Adherence to the protocol ensured that data management and quality assurance procedures aligned with the standards set forth by the data and safety monitoring guidelines (Abajobir *et al*., 2021). The survey tool was administered using tablets and programmed in SurveyCTO. Prior to the actual data collection, the tool was pre-tested in informal settlements in Nairobi and selected villages in Kakamega. Trained team leaders supervised real-time data collection in the field and conducted regular spot-checks and sit-ins on ∼5–10% of each field worker’s interviews. Before the data were sent to the central database, team leaders edited and validated the quality. The data collection tool facilitated on-the-spot quality control checks, and an automated programme ensured data completeness, correctness and consistency.

All interviews were done in private spaces and confidentiality of the data was ensured by removing identifying information contained in any data or reports. During data analysis, participants were given a unique code, and all their personal information was shredded or deleted upon completion of the data collection.

### Data analysis

The analysis was based on the baseline, midline and endline surveys, with analysis samples defined per category of outcome variable. The analyses of ‘RMNCH utilization’ focused on women aged 15–49 years at baseline. Women were included in the pre-treatment cross-section if they had completed a pregnancy with a live birth in the 3 years prior to the baseline survey, with data on their most recent live birth. Women were included in the post-treatment cross-section if they had had a live birth in the 21 months between baseline and endline (measured either in the midline survey or in the endline survey), again with data on their most recent live birth. Women were excluded if they did not have a live birth but instead a miscarriage (*n* = 5), abortion (*n* = 2) or stillbirth (*n* = 2). The analyses of ‘family planning’ outcomes included all women aged 15–49 years at baseline, prospectively using their responses from the three survey waves: baseline, midline and endline. Data on ‘unmet need’ for family planning was available at baseline and midline only. The analysis of ‘preventive services’ used cross-sectional responses from the three survey waves (baseline, midline and endline), including all children aged 9 to 59 months in each wave for vaccination outcomes, and all children aged 0 to 59 months in each wave for bed net use. Analyses only included observations with complete information on all outcome variables and covariates.

The analysis utilized both descriptive and analytical methods to analyse the data, employing the statistical software package Stata 16.1. Descriptive, bivariate analyses were conducted for each indicator of RMNCH service utilization and all covariates to compare individual-level outcomes and covariates across the treatment and control groups at baseline using a Wald test.

The multivariate analysis was initiated by examining the impact of the intervention in the control and treatment groups, with the individual women or children serving as the unit of analysis. The preferred model specification was a simple linear difference-in-difference model (DID), or a linear probability model for binary outcomes. The DID model accounts for any potential selection bias due to observable or time-invariant unobservable factors by focusing on changes in outcomes, rather than levels observed after the interventions. The model was specified as follows for outcome *Y*_ij_ using three waves of data:


(1) $${{\mathrm{Y}}_{{\mathrm{ij}}}}{\mathrm{ = }}{{{\beta }}_{\mathrm{0}}}{\mathrm{ + }}{{{\beta }}_{\mathrm{1}}}{\mathrm{R}}{{\mathrm{1}}_{\mathrm{i}}}{\mathrm{ + }}{{{\beta }}_{\mathrm{2}}}{\mathrm{R}}{{\mathrm{2}}_{\mathrm{i}}}{\mathrm{ + }}{{{\beta }}_{\mathrm{3}}}{{\mathrm{I}}_{\mathrm{i}}}{\mathrm{ + }}{{{\beta }}_{\mathrm{4}}}{\mathrm{R}}{{\mathrm{1}}_{\mathrm{i}}}{\mathrm{ \times }}{{\mathrm{I}}_{\mathrm{i}}}{\mathrm{ + }}{{{\beta }}_{\mathrm{5}}}{\mathrm{R}}{{\mathrm{2}}_{\mathrm{i}}}{\mathrm{ \times }}{{\mathrm{I}}_{\mathrm{i}}}{\mathrm{ + }}{{{\beta }}_{\mathrm{6}}}{{\mathrm{X}}_{\mathrm{i}}}{\mathrm{ + }}{{\boldsymbol{\varphi }}_{\mathrm{i}}} + { \in _{it}}\,$$

where ${Y_{i,j}}$ is the outcome $j$ for woman ${\mathrm{or child }}i$, $R{1_i}$ and $R{2_i}$ are dummy variables for the midline and endline observations, respectively, ${I_i}$ is a dummy variable taking a value of $1$ if the individual’s village was assigned to the treatment group, and 0 otherwise. The models also included covariates (β_6_X_i_) as described above and matched-pair fixed effects (**φ_i_**). The error term, ${ \in _i}$, assumed to be standard normal, was clustered at the pair-level level (de Chaisemartin and Ramirez-Cuellar, 2020). The main analysis used a ‘repeated cross-section’ sample to ensure a sufficient sample size. The coefficients ${\beta _4}$ and ${\beta _5}$ are the coefficients of interest: they represent the DID estimates that estimate the difference in the changes between the control and treatment groups at midline and endline, respectively, compared to the baseline. In other words, the DID estimate measures whether the pre-/post-treatment change in the outcome of interest is different in the two intervention groups.

As a robustness check, we also employed DID models that analysed the outcome variables using a balanced panel of samples rather than the repeated cross-section. These models represent the analysis of a dataset where observations were available for all the three waves for both the treatment and control groups. These models use the same specification as in equation (1), but restricted the sample to those observations that were available in all waves. Moreover, we applied an Analysis of Covariance (ANCOVA) model for outcome variables with a baseline value at the respondent level to control for any baseline differences and to increase the precision of our estimates from the DID model, as ANCOVA models are more robust in the presence of measurement error in the outcome variables (Egbewale *et al*., 2014), using the following specification:


(2) $${{\mathrm{Y}}_{{\mathrm{ij}}}}{\mathrm{ = }}{{{\beta }}_{\mathrm{0}}}{\mathrm{ + }}{{{\beta }}_{\mathrm{1}}}{\mathrm{R}}{{\mathrm{2}}_{\mathrm{i}}}{\mathrm{ + }}{{{\beta }}_{\mathrm{2}}}{{\mathrm{I}}_{\mathrm{i}}}{\mathrm{ + }}{{{\beta }}_{\mathrm{3}}}{\mathrm{R}}{{\mathrm{2}}_{\mathrm{i}}}{\mathrm{ \times }}{{\mathrm{I}}_{\mathrm{i}}}{\mathrm{ + }}{{{\beta }}_{\mathrm{4}}}{{\mathrm{Y}}_{{\mathrm{ij,round = 0}}}}{\mathrm{ + }}{{{\beta }}_{\mathrm{5}}}{{\mathrm{X}}_{\mathrm{i}}}{\mathrm{ + }}{{\boldsymbol{\varphi }}_{\mathrm{i}}} + { \in _{it}} $$

This specification was estimated on the midline and endline data only, while controlling for the baseline value of the outcome (${Y_{i,j,{\ }round = 0}}$). In this specification, ${\beta _2}$represents the midline impact and ${\beta _3}$ the endline impact. All other parameters were as specified for equation (1).

### Ethics approval and consent to participate

The study protocol was reviewed and approved by Amref Health Africa’s Ethical and Scientific Review Committee (P679-2019), and an amendment was granted for the move to telephone interviews and an extension of field work until the end of June 2021 due to the COVID-19 pandemic in April 2020. A research permit was obtained from Kenya’s National Commission for Science, Technology and Innovation. The data collection team and all researchers working on this study received research ethics training. Participants were given an informed consent form that explained the research and were asked for their permission to participate in the research study and data collection. Informed consent was provided before randomization. Participants were aware that they had the freedom to choose to participate in the study and that they had the right to withdraw. The permission papers were administered by field workers to those who qualified, including girls under the age of 18 who were pregnant or had a child, as they qualify as ‘emancipated minors’ (able to give their own consent). Supplementary file 2 (see supplementary online material) presents details of the methods and other sections based on CONSORT guidelines for cluster-RCT studies (Butcher *et al*., 2022).

## Results

### Individual completed surveys

Table 1 provides an overview of the study samples used in the analyses for each of the outcome categories, as described in the Data analysis section above. For the analysis of RMNCH utilization, we used a sample of 248 women with a live birth either within the past 3 years at baseline and/or between baseline and endline. A total of 163 women reported a live birth in the 3 years prior to the baseline survey, and 185 women reported a live birth between baseline and endline (182 of them reported the live birth at midline, and another three women reported it at endline). The births registered at midline or endline were merged into one post-treatment cohort—there is no double-counting in this cohort. In total, 95 women recorded a recent live birth both pre-treatment (baseline) as well as post-treatment (midline/endline). The study sample for assessing family planning outcomes consisted of 346 women, 191 of whom were observed across all three waves of data collection. Data on unmet need was available for 266 married/partnered women aged 15–49 years old with 212 observations at baseline and 220 observations at midline. The analysis of vaccination outcomes included a total of 398 children, with 254 observations at baseline, 291 at midline and 267 at endline. In total, 132 children were observed across all three waves of data collection (balanced panel). For the assessment of bednet use, a sample of 424 children was included in the analysis, of whom 165 were observed in all three waves.

**Table 1.** Study participants per survey wave

	Women			Children	
Survey wave	RMNCH healthcare utilization	Family planning	Unmet need	Vaccination	Bednet use
Baseline	163	280	212	254	297
Midline		271	220	291	322
Endline	185 (182 + 3)	248		267	283
Balanced in all three waves	95	191	166	132	165
Number of unique observations	248	346	266	398	424

### Baseline description of the study sample

Table 2 shows the baseline means of our main variables of interest in the treatment and control groups (see supplementary Table SA1 for means at midline and endline in the supplementary online material). To examine potential imbalance in baseline outcomes and covariates, separate balance tests were conducted for the baseline means of all variables by regressing the baseline outcome (or covariate) on a treatment indicator. The findings (Table 2 final column) demonstrated that the differences between the treatment and control group at baseline were not statistically significant, except for one variable (short-acting contraceptive use, *P* = 0.054). This is to be expected, given the random assignment of communities to treatment arms. It shows that the randomization process was effective.

**Table 2.** Outcomes by baseline sociodemographic and economic characteristics

	Control		Treatment		Mean	Difference	
Variable	Mean	N1	Mean	N2	Difference	SE	*P*-value
**PANEL A. RMNCH health care utilization**							
ANC visits	0.98	66	1.00	97	0.02	0.02	0.36
At least four ANC visits	0.67	66	0.78	97	0.12	0.07	0.11
Tetanus toxoid injection at ANC	0.59	66	0.63	97	0.04	0.11	0.75
Iron supplements at ANC	0.97	66	0.96	97	−0.01	0.02	0.64
Folic acid at ANC	0.94	66	0.91	97	−0.03	0.04	0.41
Malaria prevention during ANC	0.95	66	0.98	97	0.02	0.04	0.52
Quality of ANC was good/very good	0.89	65	0.85	97	−0.05	0.07	0.49
Delivery in health facility	0.91	66	0.96	97	0.05	0.05	0.35
Delivery by SBA	0.88	66	0.94	97	0.06	0.05	0.24
Quality of delivery services was good/very good	0.86	66	0.89	97	0.02	0.03	0.51
Immediate PNC	0.83	66	0.75	97	−0.08	0.10	0.42
PNC check-up after discharge	0.15	66	0.16	97	0.01	0.06	0.83
PNC received within 2 days	0.06	66	0.02	97	−0.04	0.04	0.31
PNC for baby	0.50	66	0.42	97	−0.08	0.08	0.33
**Covariates**							
Age (in years) of individual	28.39	66	28.64	97	0.25	0.83	0.77
Household in lowest wealth tercile	0.38	66	0.37	97	−0.01	0.08	0.92
Household in middle wealth tercile	0.38	66	0.29	97	−0.09	0.07	0.20
Household in highest wealth tercile	0.24	66	0.34	97	0.10	0.07	0.20
Individual can read and write in any language	0.94	66	0.91	97	−0.03	0.03	0.34
**PANEL B. Family planning use**							
Any modern contraceptive	0.45	137	0.50	143	0.04	0.06	0.49
Short-acting contraceptive	0.17	137	0.28	143	0.11	0.05	0.05*
Long-acting contraceptive	0.28	137	0.22	143	−0.06	0.05	0.22
Unmet need for family planning	0.32	100	0.27	112	−0.05	0.06	0.37
**Covariates**							
Age (in years) of individual	28.74	137	29.20	143	0.47	1.10	0.68
Household in lowest wealth tercile	0.35	137	0.33	143	−0.02	0.06	0.72
Household in middle wealth tercile	0.38	137	0.29	143	−0.09	0.06	0.15
Household in highest wealth tercile	0.27	137	0.38	143	0.11	0.08	0.18
Individual can read and write in any language	0.93	137	0.92	143	−0.02	0.02	0.36
**PANEL C. Bednet use**							
Child slept under bednet	0.84	142	0.85	155	0.01	0.04	0.86
**Covariates**							
Age (in years) of individual	1.88	142	1.75	155	−0.13	0.10	0.19
Sex (male)	0.57	142	0.53	155	−0.04	0.04	0.35
Household in lowest wealth tercile	0.38	142	0.37	155	−0.01	0.07	0.86
Household in middle wealth tercile	0.34	142	0.28	155	−0.06	0.07	0.42
Household in highest wealth tercile	0.28	142	0.35	155	0.07	0.08	0.38
**PANEL D. Vaccination**							
Child fully vaccinated	0.80	124	0.80	130	0.00	0.07	0.98
Number of vaccinations (0–9)	8.52	124	8.56	130	0.04	0.24	0.88
**Covariates**							
Age (in years) of individual	2.15	124	2.08	130	−0.07	0.13	0.60
Sex (male)	0.56	124	0.54	130	−0.02	0.05	0.72
Household in lowest wealth tercile	0.39	124	0.35	130	−0.03	0.07	0.63
Household in middle wealth tercile	0.35	124	0.28	130	−0.06	0.07	0.42
Household in highest wealth tercile	0.27	124	0.36	130	0.10	0.09	0.33

Notes: *P*-values are reported from Wald tests on the equality of means of treatment and comparison for each variable. N1 and N2 show the total number of observations per variable in the control and treatment arm, respectively. Standard errors (SE) are clustered at the pair level.

* *P *< 0.1.

Table 2 panel A shows that nearly all women reported at least one ANC visit for their most recent live birth (97–100%) and a substantial share of women received four or more ANC visits (67–78%). Out of four common ANC services, a tetanus toxoid injection was least common (59–63%), while iron supplements, folic acid and malaria prevention measures were all reported by >90% of women. Between 85–89% of women rated their experience with the ANC services as ‘good’ or ‘very good’. The large majority of women delivered their most recent live birth in a health facility (91–96%) and with the assistance of an SBA (88–94%). Between 86–89% of women rated the delivery services ‘good’ or ‘very good’. The receipt of immediate PNC services was equally high at 75–83%. However, only 15–16% of women received a PNC check-up after discharge and a very small share of women (2–6%) received a PNC check-up within 2 days after discharge. In addition, less than half (42–50%) of newborns received a PNC check-up.

Table 2 panel B shows that 45–50% of women aged 15 to 49 used some form of modern contraceptive, with the rate for short-acting contraceptives being lower in the control group at 17% compared to 28% in the treatment group (*P* = 0.054). The share of women using long-acting contraceptives was slightly higher in the control group (28%) than in the treatment group (22%) but not significantly different. Between 27–32% of women exhibited an unmet need for family planning at baseline. We observed 84–85% of children 0–59 months old sleeping under a bednet the day before the survey (Table 2 panel C). Among children aged 9–59 months, 80% had been fully vaccinated, with an average number of ∼8.5 vaccinations out of the nine recommended shots (Table 2 panel D).

Supplementary Table SA2 (see supplementary online material) describes general trends in outcomes over time in the study area, during the peak of the COVID-19 pandemic, showing increases of 10 percentage point (pp) in attaining at least four ANC visits, 14 pp in tetanus-toxoid vaccinations, 7 pp in folic acid and 6 pp in skilled deliveries. The use of any modern contraceptives increased by 14 pp at endline, driven by an increase of 19 pp in long-acting contraceptives, leading to a decrease of 12 pp in unmet need for family planning at midline. Vaccination rates were not statistically different at endline, but children were 12 pp more likely to sleep under a bed net.

### Impact of i-PUSH on RMNCH services utilization

Tables 3–5 present the DID findings of the impact of *i*-PUSH on RMNCH healthcare utilization, family planning uptake and preventive services for children, respectively. There was no statistically significant effect at the 5%-significance level on any of the outcome indicators in the DID models. Table 3 describes the impact estimates on ANC, SBA and PNC outcomes. All impacts on ANC-related outcomes are statistically insignificant, including the number of ANC visits and whether the mother received a tetanus toxoid injection, iron supplements, folic acid or malaria prevention during ANC. The impact estimates for delivery at a health facility are negative and significant at the 10%-level, capturing a smaller increase in deliveries at health facilities of 9.6 pp in the treatment group compared to the control group. In line with this result, the change in the fraction of births attended by a skilled attended was 7.7 pp lower in the treatment group than in the control group. Finally, none of the PNC-related estimates are statistically significant.

**Table 3.** Impact of *i*-PUSH on RMNCH healthcare utilization

		Baseline treated	Baseline control	Endline treated	Endline control
	Impact estimate	mean	mean	mean	mean
Outcome variable	(1)	(2)	(3)	(4)	(5)
Any ANC visits	−0.016	1.000	0.985	1.000	1.000
	(0.02)				
At least four ANC visits	−0.086	0.784	0.667	0.854	0.830
	(0.06)				
Tetanus toxoid injection at ANC	−0.121	0.629	0.591	0.708	0.807
	(0.11)				
Iron supplements at ANC	0.071	0.959	0.970	0.917	0.864
	(0.12)				
Folic acid at ANC	0.015	0.907	0.939	0.990	1.000
	(0.04)				
Malaria prevention during pregnancy	−0.067	0.979	0.955	0.948	0.989
	(0.05)				
Quality of ANC was good/very good	0.071	0.845	0.892	0.917	0.898
	(0.08)				
Delivery in health facility	−0.096*	0.959	0.909	0.948	0.989
	(0.05)				
Delivery with SBA	−0.077*	0.938	0.879	0.969	0.977
	(0.04)				
Quality of delivery services was good/very good	−0.052	0.887	0.864	0.823	0.864
	(0.05)				
Immediate PNC	0.109	0.753	0.833	0.875	0.852
	(0.13)				
PNC check-up after discharge	−0.100	0.165	0.152	0.135	0.205
	(0.09)				
PNC received within 2 days	−0.028	0.021	0.061	0.042	0.114
	(0.04)				
PNC for baby	0.120	0.423	0.500	0.594	0.568
	(0.11)				
***n***	**347**	**97**	**66**	**96**	**88**

Standard errors in parentheses are clustered at the randomization pair-level.

* *P *< 0.1.

Estimations use DID modelling and include the following covariates: woman’s age, woman’s literacy, household wealth and pair fixed effects. Cross-sectional samples consist of all women aged 15–49 years at baseline with a live birth in the 3 years prior to the baseline survey (baseline) and/or a live birth in the 21 months between baseline and endline (endline).

Note: skilled birth attendant: any of doctor, nurse midwife; unskilled: all others, including community health workers/traditional birth attendants. Facility delivery: giving birth in a healthcare facility maternity/neonatal care.

Table 4 shows the results for the family planning analyses, with the impact results at midline and endline shown in columns (2) and (3), respectively. We did not find a statistically significant impact for any modern contraceptive, nor for short-acting and long-acting contraceptives separately. These findings hold both at midline and endline, and estimates are not significantly different from each other over time (column 4). The midline impact on unmet need for family planning in the treatment group is not significant either.

**Table 4.** Impact of *i*-PUSH on family planning (repeated cross-section of samples)

		Control mean,	Impact:	Impact	Impact diff
		baseline	midline	endline	(endline vs midline)
Outcome variable	No. of Obs.	(1)	(2)	(3)	(4) = (3) − (2)
Any modern contraceptive	798	0.453	−0.035	0.039	0.074
			(0.08)	(0.10)	(0.05)
Short-acting contraceptive	798	0.168	−0.044	−0.030	0.014
			(0.07)	(0.08)	(0.06)
Long-acting contraceptive	798	0.285	0.024	0.068	0.044
			(0.09)	(0.11)	(0.07)
Unmet need for family planning	431	0.320	0.067		
			(0.08)		

Standard errors in parentheses are clustered at the randomization pair-level.

Estimations use DID modelling and include the following covariates: woman’s age, woman’s literacy, household wealth and pair fixed effects. The sample consists of all women aged 15–49 years at baseline, followed over time. Data for unmet need was only available at baseline and midline.

Table 5 shows the impact estimates of *i*-PUSH on preventive health indicators for children. The programme did not have a significant effect on the likelihood that children were fully vaccinated, nor on the number of vaccinations they received. The results for sleeping under a bednet are not statistically significant either. These findings apply to the impact analyses at midline as well as at endline.

**Table 5.** Impact of *i*-PUSH on vaccination uptake and bednet use among children

		Control mean,	Impact:	Impact:	Impact difference
	No. of observations	baseline	midline	endline	(endline vs midline)
Outcome variable		(1)	(2)	(3)	(4) = (3) − (2)
Child fully vaccinated	811	0.798	−0.062	−0.074	−0.012
			(0.06)	(0.14)	(0.11)
Number of vaccinations (0–9)	811	8.524	−0.063	−0.257	−0.194
			(0.18)	(0.27)	(0.15)
Child slept under bednet	901	0.838	0.059	−0.003	−0.062
			(0.07)	(0.05)	(0.05)

Standard errors in parentheses are clustered at the randomization pair-level.

Estimations use DID modelling and include the following covariates: child’s age, child sex, household wealth and pair fixed effects. The cross-sectional samples for the vaccination estimations consist of all children aged 9–59 months at the time of the survey. The cross-sectional samples for the bednet estimation consists of all children aged 0–59 months at the time of the survey.

Note: Fully vaccination refers to the uptake of all basic vaccines (BCG, polio 0, polio 1, polio 2, polio 3, hepatitis B virus, DPT 1, DPT 2, DPT 3 and measles).

Supplementary analyses using a balanced panel of samples for all outcomes showed similar findings (supplementary Tables SA3–SA5, see supplementary online material), except for the use of long-acting contraceptives at midline, which had increased significantly more in the treatment group versus the control group. Comparable non-significant results were also found using an ANCOVA specification (supplementary Tables SA6–SA8), the only exception being the decreased probability of receiving a tetanus toxoid injection during ANC in the treatment group, which was not significant in the DID models.

## Discussion

To enhance the effectiveness of RMNCH interventions in low- and middle-income settings, the literature suggests incorporating the following components (Ferrari and Cribari-Neto, 2004; Comfort *et al*., 2013; Alam and Mahal, 2014; Warton and Parker, 2018; Aksünger *et al*., 2022): improving availability, appropriateness, affordability, approachability and acceptability of RMNCH services across the continuum of care; providing improved training for health professionals to improve the quantity and quality of professional–mother interactions so as to increase mothers’ knowledge of RMNCH and childcare; and conducting outreach at the household and community level by providing demand-side financial or knowledge support to improve healthcare access and reduce inequities. The *i*-PUSH programme is a mobile-based insurance initiative that addresses many of these aspects. The programme seeks to improve the availability and appropriateness of good quality care by upgrading selected healthcare facilities. Improving the approachability and acceptability of care as well as knowledge in the community was achieved by providing digital training to CHVs on specific health areas of interest and health insurance. Finally, affordability was enhanced by the provision of fully subsidized NHIF insurance for the first year.

This evaluation focused on the impact of health insurance, conditional on the implementation of the other components of the *i*-PUSH programme, which were implemented in the entire study area. We employed a cluster-RCT design to isolate the demand-side effect of subsidized, digital insurance, relieving logistical and administrative barriers through hands-on enrolment support. Villages in the catchment areas of four upgraded facilities were randomly assigned either to the treatment group, receiving free insurance after baseline, or to the control group, receiving free insurance after endline. Households in both groups had access to improved knowledge and quality of care, but only treatment households could access care at highly reduced costs. A companion study that evaluates the impact of *i*-PUSH insurance coverage on healthcare utilization, unrelated to RMNCH, shows a positive impact on financial protection and treatment of illnesses and injuries of both adults and children, especially for households living close to the facilities (de Groot *et al*., 2023).

Our findings indicate that the expanded choice and coverage of NHIF did not result in increased utilization of RMNCH services. We observed no significant differences between the treatment and control groups on most outcome variables. The notable exception is the likelihood of delivering in a health facility and in the presence of an SBA. This likelihood increased more strongly in the control communities over time compared to the treatment communities, yielding a negative programme impact estimate. This effect is likely driven by ceiling effects, however (with SBA rates of 97% in both groups at endline), in combination with slightly—albeit insignificantly—lower baseline values in the control group (88%) compared to the treatment group (94%). Thus, the fact that the treatment group had less room for improvement to begin with might have led to the negative impact estimates.

One possible reason for the lack of impact of the health insurance component of *i*-PUSH is the limited added value it provided on top of Linda Mama, a national programme that offers free selected maternal care services at public facilities. Linda Mama may have been sufficient to relieve pregnant women in the study area of most barriers to seeking maternal care, including ANC, deliveries and PNC services. The NHIF SupaCover benefit package is more encompassing, unlimited in time and provides a greater choice of (primary, secondary, tertiary public as well as private) providers than what is covered under Linda Mama. Despite expectations, these additional benefits do not seem to have affected the uptake of RMNCH services in a substantive way. Oparanya care, another public RMNCH programme, was also active in the study area, potentially further diluting any positive impacts of the insurance scheme on maternal care utilization.

Nevertheless, in the descriptive analyses, discernible positive shifts were observed for several outcome variables within intervention villages, albeit without attaining statistical significance regarding the anticipated impact. A second reason for the lack of significant impacts may hence be due to the limited power of the study to pick up modest changes in secondary outcomes. Thus, while we can conclude that the programme did not yield sizable impacts, we cannot rule out that it produced more subtle effects on the target population.

A third explanation of the limited findings relates to the other *i*-PUSH components, i.e. the quality upgrades at selected facilities and the digital training of CHVs, which affected all households in the study area—regardless of whether they were living in treatment or control villages. To the extent that these components also contributed to enhanced RMNCH utilization, this may have masked any partial effects of the subsidized insurance coverage. The programme was implemented during the outbreak of the COVID-19 pandemic, a period in which economic conditions and access to transport generally worsened and access to RMNCH services stagnated (Janssens *et al*., 2021; Gómez-Pérez *et al*., 2023). In Kenya, women reported reduced access to basic health services due to the pandemic’s lockdown and curfew, leading to fear of contracting the virus, de-prioritization of health services, economic constraints and psychosocial effects. The World Health Organization (WHO) has also highlighted the impact of the pandemic on the provision and use of RMNCH interventions (Oluoch-Aridi *et al*., 2020; WHO, 2021). Despite these challenges, our study area showed significant improvements in many RMNCH outcomes at endline compared to baseline. This provides suggestive evidence that the full combination of intervention components was effective in keeping women of reproductive age and their children onboarded in the health system. Since the quality improvement plans and enhanced CHV training were implemented throughout the study area, they could not be evaluated in our impact study.

One strength of this study is the innovativeness of the *i*-PUSH programme which adopts mobile-based platforms, facilitating enrolment in health insurance and providing tailored training to CHVs to improve access to upgraded RMNCH care. Rigorous evaluations of such innovative mHealth approaches, which simultaneously address both demand- and supply-side constraints, are scarce (Bonfrer *et al*., 2018).

However, the evaluation also shows some limitations. First, as discussed above, the existing coverage of national maternal care programmes (e.g. Linda Mama and Oparanya Care), may have diminished the added value for RMNCH outcomes of providing more comprehensive health insurance. Furthermore, the inability to evaluate all programme components hampers a comprehensive assessment of effectiveness, leading us to focus on the insurance component only. A third limitation is the relatively small sample size which restricts our ability to pick up modest impacts on secondary outcomes. Fourth, the COVID-19 pandemic had a detrimental effect on health outcomes throughout the country. The significant positive time trend that we observed between baseline and endline, with substantial increases in the uptake of RMNCH services during the height of the pandemic across all study communities, suggests that *i*-PUSH might have cushioned the worse effects of COVID-19 and might have yielded stronger impacts in normal times. Fifth, using self-reported outcome measures might have introduced recall, social desirability and misclassification biases. Finally, any differences between the private and public facility upgraded by *i*-PUSH, such as efficiency and service quality, may have influenced the intervention’s impact across communities. However, since their catchment areas included both treatment and control households, we do not expect heterogeneous impact estimates to have been biased by such provider differences. We expect our results to be generalizable to other rural communities in Kenya as well as to other east African countries more broadly, where RMNCH is included in basic public health care, enrolment in health insurance is mostly confined to formal sector workers, the use of mobile phones is widespread and COVID-19 had a major impact on access to care.

We conclude that the inclusion of RMNCH services in the comprehensive NHIF SupaCover package carried little added value as key services were already covered under the—more basic but universal—Linda Mama programme. The positive trends suggest, however, that the combination of enhanced quality of care and improved CHV training, from which both control and treatment groups benefited, had a beneficial impact on usage of RMNCH services, at a time when access to healthcare was severely under pressure due to the COVID-19 pandemic.

## Supplementary Material

czae079_Supp

## Data Availability

All data generated or analysed during this analysis are included in the submitted manuscript (and its supplementary files).

## Abbreviations

ANC = antenatal care

ANCOVA = analysis of covariance

BCG = Bacillus Calmette-Guérin

CHVs = community health volunteers

CONSORT = Consolidated Standards of Reporting Trials

DID = difference-in-difference

DPT = diphtheria, pertussis, and tetanus

*i*-PUSH = Innovative Partnership for Universal Sustainable Healthcare

MDE = minimum detectable effect

mHealth = mobile health

RMNCH = reproductive, maternal, newborn and child healthcare

NHIF = National Hospital Insurance Fund

PNC = postnatal care

PP = percentage point

RCT = randomized controlled trial

SBA = skilled birth attendant

SSA = sub-Saharan Africa

## Reflexivity statement

This manuscript involved collaboration between six researchers from Kenya and The Netherlands, with three of them being senior researchers, of whom two were female. All authors possess interdisciplinary expertise in public health, medicine and social sciences. This collective effort has contributed to the comprehensive approach taken in this work.

## Appendix 1. Description of the i-PUSH program

This additional text provides a concise summary of the intervention using the TIDIER framework (Hoffmann *et al*., 2014) (see supplementary file 1 for more information). *i*-PUSH provides the NHIF SupaCover for free to a low-income population, low-income women and their family members, in Western Kakamega County and in Nairobi. To improve access to inexpensive and high-quality health care, the programme makes use of novel digital solutions developed by Amref Health Africa and the PharmAccess Foundation, with the aim of increasing access to quality healthcare services. Through PharmAccess’ SafeCare quality enhancement programme, selected facilities in the programme areas participate in a quality assessment and develop a quality improvement plan. The programme’s first point of contact, CHVs, employ Amref Health Africa’s Mjali (Mobile Jamii Afya Link) tool for digital registration of household information and Amref’s mobile phone-based LEAP tool for training. LEAP uses a mobile learning strategy to train and empower CHVs utilizing their mobile devices, which can run on any phone and allow them to learn at their own pace while being in the community, giving both interpersonal and community aspects of learning (Abajobir *et al*., 2021). Through the use of the LEAP training tool, the programme enhances the capacity of CHVs, and through the knowledge they receive, the CHVs encourage and support women to visit the upgraded clinics. Finally, using PharmAccess ‘health wallet’, women also obtain the NHIF insurance card for free on their mobile phone via the *i*-PUSH programme. The ‘health wallet’ is powered by M-TIBA, a digital platform, and it tracks their health care utilization at the designated facilities, making patient tracking and doctor–patient contact easier. M-TIBA facilitators support women in registration on NHIF by helping them to fill out the required documentation and uploading the necessary documents.

Moreover, the *i*-PUSH programme offers an additional feature that allows women of reproductive age and their household members, including spouses, children and dependents, to enrol in health insurance digitally. CHVs can use their smartphones, provided by the programme, to upload the required documents and complete the registration process during their visits to the households, as long as the women have an ID or birth certificate available for their children. This process eliminates the need for women to repeatedly visit insurance offices and submit all the necessary paperwork, reducing the logistical and time barriers to insurance adoption.

Overall, besides reducing out-of-pocket health expenditure, the digital insurance offers several potential pathways to enhance RMNCH outcomes. For instance, by equipping CHVs with improved skills to educate on RMNCH and insurance, it fosters increased dialogue within communities and enhances knowledge among women and men regarding RMNCH and financial mechanisms This, in turn, cultivates positive attitudes towards insurance and health savings, leading to greater financial preparedness for health expenses among women of reproductive age. As a result, women of reproductive age may exhibit increased health-seeking behaviour and utilize RMNCH services more regularly, ultimately contributing to improved RMNCH outcomes.

## Appendix 2. Study design

A matched-pair longitudinal cluster-RCT was employed. To ensure the robustness of the cluster-RCT, the procedure for matching control and treatment villages followed three steps: (1) the random selection of 24 villages, (2) the pair-matching of villages based on relevant background characteristics and baseline outcomes of interest, stratified by health facility catchment area and (3) the randomization of villages within each pair into treatment or control by flipping a coin. Randomization at the village level was conducted during a public ceremony in Khwisero Subcounty. Control and treatment groups were formed by randomly assigning villages to either the treatment group, where *i*-PUSH was implemented after the baseline survey, or the control group, where it was not implemented until after the endline survey. To enhance the probability of achieving two well-balanced comparable groups, villages were paired before randomization. Randomization without pairing could result in control and treatment groups with significantly different characteristics due to chance. To create pairs of similar villages and determine exact matching indicators, baseline data and community-level socio-demographic and infrastructure indicators (including the number of households, access to basic amenities and public services at the village level, adult literacy rate, women’s literacy rate, perceived health status and healthcare utilization indicators) were used by the research team. The ‘Euclidean distance’ method was employed for the matching procedure, which calculates the absolute difference between the standardized values of all covariates for a given pair of matches. Matching was done separately for each of the four originally chosen health centre catchment areas. Each village was paired with one of the other five villages near the health centre to ensure an equal number of control and treatment villages in each catchment area. The Euclidean distance was calculated between each village and all other villages within the same catchment area. The two villages with the shortest distance were paired, removed from the list, and the distance calculation was repeated, excluding the newly paired villages, until all villages were paired.

After matching, the randomization assignment was conducted in the presence of important stakeholders, such as the PharmAccess Foundation’s area manager, local liaison individuals and village leaders. Local government officials provided their consent to the methods before the random assignment. The steps that were taken were: paired village names were written on papers, folded and put into a bag; two representatives from each paired village discussed who would pick the paper, and after other group members verified that the names were not visible, one paper was chosen. The chosen village was then assigned to a group by flipping a 10 Kenyan Shilling coin. Village leaders decided that the head of the coin would represent the control group and the shield would represent the treatment group, using Kenyatta (first president of Kenya) village as an example of a ‘controlling’ village. The process of selecting the folded paper and flipping the coin was repeated for all paired villages. The *i*-PUSH programme introduced the subsidized health insurance to the treatment communities between June and October 2020. CHVs, both in the treatment and the control communities, received training on LEAP from October 2020 onwards.

## Appendix 3. Ex-post power calculations for RMNCH indicators

**Table:**

Indicator	Baseline value	MDE	% Change	Sample size
**Any modern contraceptive**	0.475	0.166	0.35	280
Short-acting contraceptive	0.225	0.181	0.80	280
Long-acting contraceptive	0.254	0.159	0.63	280
Unmet need for family planning	0.292	0.187	0.64	212
**At least four ANC visits**	0.736	0.183	0.25	163
Tetanus toxoid injection at ANC	0.613	0.308	0.50	163
Quality of ANC was good/very good	0.864	0.124	0.14	162
Quality of delivery services was good/very good	0.877	0.110	0.13	163
**Immediate PNC**	0.785	0.192	0.24	163
PNC check-up after discharge	0.160	0.254	1.59	163
PNC received within 2 days	0.037	0.209	5.65	163
PNC for baby	0.454	0.293	0.65	163
**Child fully vaccinated**	0.799	0.148	0.19	254
Number of vaccinations (0–9 months)	8.543	0.723	0.08	254
Child slept under bednet	0.842	0.111	0.13	297

## References

[R1] Abajobir A, de Groot R, Wainaina C et al. 2021. The impact of *i*-PUSH on maternal and child health care utilization, health outcomes, and financial protection: study protocol for a cluster randomized controlled trial based on financial and health diaries data. *Trials* 22: 629. 10.1186/s13063-021-05598-7 PMC8443110 34526072

[R2] Aker JC, Isaac MM. 2010. Mobile phones and economic development in Africa. *Journal of Economic Perspectives* 24: 207–32.

[R3] Aksünger N, De Sanctis T, Waiyaiya E et al. 2022 What prevents pregnant women from adhering to the continuum of maternal care? Evidence on interrelated mechanisms from a cohort study in Kenya. *BMJ Open* 12: e050670. 10.1136/bmjopen-2021-050670 PMC8765038 35039285

[R4] Alam K, Mahal A. 2014. Economic impacts of health shocks on households in low and middle income countries: a review of the literature. *Globalization & Health* 10: 21. 10.1186/1744-8603-10-21 PMC4108100 24708831

[R5] Alpay LL, Anne O, Henkemans B, Otten W, Dumaij ACM. 2010. E-health applications for patient empowerment in the Netherlands: directions for best practices in the Netherlands. *Telemedicine and e-Health* 16: 787–91. 20815745 10.1089/tmj.2009.0156

[R6] Amref Health Africa . 2020. Leap, the mHealth platform. https://amref.org/enterprises/our-products/leap/, accessed 3 February 2023.

[R7] Aranda-Jan CB, Mohutsiwa-Dibe N, Loukanova LS. 2014. Systematic review on what works, what does not work and why of implementation of mobile health (mHealth) projects in Africa. *BMC Public Health* 14: 1–15. 24555733 10.1186/1471-2458-14-188 PMC3942265

[R8] Benkeser D, Díaz I, Luedtke A et al. 2021. Improving precision and power in randomized trials for COVID-19 treatments using covariate adjustment, for binary, ordinal, and time-to-event outcomes. *Biometrics* 77: 1467–81. 32978962 10.1111/biom.13377 PMC7537316

[R9] Bonfrer I, Van de Poel E, Gustafsson-Wright E, Van Doorslaer E. 2018. Voluntary health insurance in Nigeria: effects on takers and non-takers. *Social Science & Medicine* 205: 55–63. 29653298 10.1016/j.socscimed.2018.03.035

[R10] Bradley SEK, Croft NT, Fishel JD, Westoff CF. 2012. Revising unmet need for family planning. DHS analytical atudies No. 25. Calverton, Maryland, USA: ICF International. https://dhsprogram.com/pubs/pdf/AS25/AS25%5B12June2012%5D.pdf, accessed 23 February 2023.

[R11] Butcher NJ, Monsour A, Mew EJ et al. 2022. SPIRIT-Outcomes 2022 extension: guidelines for reporting outcomes in trial protocols. *Jama* 328: 2345–56. 36512367 10.1001/jama.2022.21243

[R12] Comfort AB, Peterson LA, Hatt LE. 2013. Effect of health insurance on the use and provision of maternal health services and maternal and neonatal health outcomes: a systematic review. *Journal of Health, Population and Nutrition* 31: 81–105.24992805

[R13] Crawford J, Larsen-Cooper E, Jezman Z, Cunningham SC, Bancroft E. 2014. SMS versus voice messaging to deliver MNCH communication in rural Malawi: assessment of delivery success and user experience. *Global Health: Science and Practice* 2: 35–46. 25276561 10.9745/GHSP-D-13-00155 PMC4168599

[R14] de Chaisemartin C, Ramirez-Cuellar J. 2020. At what level should one cluster standard errors in paired experiments, and in stratified experiments with small strata? National Bureau of Economic Research. https://www.nber.org/system/files/working_papers/w27609/w27609.pdf, accessed 3 March 2023.

[R15] de Groot R, Abajobir A, Wainaina C et al. 2023. The impact of *i*-PUSH on low-income women UHC, OOPs. MedRxiv preprint posted July 8.

[R16] Duku SKO, Nketiah-Amponsah E, Janssens W, Pradhan M, Puebla I. 2018. Perceptions of healthcare quality in Ghana: does health insurance status matter?. *PloS One* 13: e0190911. 10.1371/journal.pone.0190911 PMC5770037 29338032

[R17] Egbewale BE, Lewis M, Sim J. 2014. Bias, precision and statistical power of analysis of covariance in the analysis of randomized trials with baseline imbalance: a simulation study. *BMC Medical Research Methodology* 14: 49. 10.1186/1471-2288-14-49 PMC3986434 24712304

[R18] Fadlallah R, El-Jardali F, Hemadi N et al. 2018. Barriers and facilitators to implementation, uptake and sustainability of community-based health insurance schemes in low- and middle-income countries: a systematic review. *International Journal for Equity in Health* 17: 13. 10.1186/s12939-018-0721-4 PMC5789675 29378585

[R19] Ferrari S, Cribari-Neto F. 2004. Beta regression for modelling rates and proportions. *Journal of Applied Statistics* 31: 799–815.

[R20] Geng X, Janssens W, Kramer B, van der List M. 2018. Health insurance, a friend in need? Impacts of formal insurance and crowding out of informal insurance. *World Development* 111: 196–210.

[R21] Gómez-Pérez GP, de Groot R, Abajobir AA et al. 2023. Reduced incidence of respiratory, gastrointestinal and malaria infections among children during the COVID-19 pandemic in Western Kenya: An analysis of facility-based and weekly diaries data. *Journal of Global Health* 13: 06024. 10.7189/jogh.13.06024 PMC10346133 37448326

[R22] Hemming K, Girling AJ, Sitch AJ, Marsh J, Lilford RJ. 2011. Sample size calculations for cluster randomised controlled trials with a fixed number of clusters. *BMC Medical Research Methodology* 11: 102. 10.1186/1471-2288-11-102 PMC3149598 21718530

[R23] Hoffmann TC, Glasziou PP, Boutron I et al. 2014. Better reporting of interventions: template for intervention description and replication (TIDieR) checklist and guide. *BMJ* 348:g1687. 10.1136/bmj.g1687 24609605

[R24] Imai K, King G, Nall C. 2009. The Essential role of pair matching in cluster-randomized experiments, with application to the mexican universal health insurance evaluation. *Statistical Science* 24: 29–53.

[R25] Janssens W, Pradhan MP, de Groot R et al. 2021. The short-term economic effects of COVID-19 on low-income households in rural Kenya: An analysis using weekly financial household data. *World Development* 138: 105280.

[R26] Johnson MC, Schellekens O, Stewart J et al. 2016. SafeCare: An innovative approach for improving quality through standards, benchmarking, and improvement in low- and middle-income countries. *The Joint Commission Journal on Quality and Patient Safety* 42: 350–P11. 27456416 10.1016/s1553-7250(16)42049-0

[R27] Kannisto KA, Koivunen MH, Välimäki MA. 2014. Use of mobile phone text message reminders in health care services: a narrative literature review. *Journal of Medical Internet Research* 16: e222. 10.2196/jmir.3442 PMC4211035 25326646

[R28] Kenya National Bureau of Statistics (KNBS) . 2022. The 2022 Kenya Demographic and Health Survey (2014 KDHS). https://dhsprogram.com/pubs/pdf/PR143/PR143.pdf, accessed 6 March 2023.

[R29] Kruk ME, Gage AD, Joseph NT et al. 2018. Mortality due to low-quality health systems in the universal health coverage era: a systematic analysis of amenable deaths in 137 countries. *The Lancet* 392: 2203–12. 10.1016/S0140-6736(18)31668-4 PMC6238021 30195398

[R30] Lara C, Liesbeth C, Joachim DW. 2017. From corn to popcorn? Urbanization and food consumption in sub-Sahara Africa: evidence from rural-urban migrants in Tanzania, LICOS Discussion Paper, No. 390. Leuven: Katholieke Universiteit Leuven, LICOS Centre for Institutions and Economic Performance. http://hdl.handle.net/10419/172042, accessed 11 March 2023.

[R31] Ngabo F, Nguimfack J, Nwaigwe F et al. 2012. Designing and implementing an innovative SMS-based alert system (RapidSMS-MCH) to monitor pregnancy and reduce maternal and child deaths in Rwanda. *Pan African Medical Journal* 13: 31. PMC3542808 23330022

[R32] Oluoch-Aridi J, Chelagat T, Nyikuri MM et al. 2020. COVID-19 effect on access to maternal health services in Kenya. *Frontiers in Global Women’s Health* 1: 599267. 10.3389/fgwh.2020.599267 PMC8593959 34816169

[R33] Onambele L, Guillen-Aguinaga S, Guillen-Aguinaga L et al. 2023. Trends, projections, and regional disparities of maternal mortality in Africa (1990-2030): An ARIMA forecasting approach. *Epidemiologia (Basel)* 4: 322–51. 37754279 10.3390/epidemiologia4030032 PMC10528291

[R34] Orangi S, Kairu A, Ondera J et al. 2021. Examining the implementation of the Linda Mama free maternity program in Kenya. *The International Journal of Health Planning and Management* 36: 2277–96. 34382238 10.1002/hpm.3298 PMC9290784

[R35] Sife AS, Kiondo E, Lyimo-Macha JG. 2017. Contribution of mobile phones to rural livelihoods and poverty reduction in Morogoro region, Tanzania. *The Electronic Journal of Information Systems in Developing Countries* 42: 1–15.

[R36] Spaan E, Mathijssen J, Tromp N et al. 2012. The impact of health insurance in Africa and Asia: a systematic review. *Bull WHO* 90: 685–92. 22984313 10.2471/BLT.12.102301 PMC3442382

[R37] UNICEF . 2015. Committing to child survival: a promise renewed. *Progress report*. https://www.unicef.org/reports/committing-child-survival-promise-renewed.

[R38] UN IGME . 2021. Levels & Trends in Child Mortality: Report 2021. United Nations Children’s Fund (UNICEF), World Health Organization (WHO), World Bank Group, and United Nations Department of Economic and Social Affairs (UN DESA). UN IGME. https://www.unicef.org/media/113251/file/UN-IGME-child-mortality-report-2021.pdf.

[R39] Wagstaff A, Flores G, Hsu J et al. 2014. Progress on catastrophic health spending in 133 countries: A retrospective observational study. *The Lancet Global Health* 6: e169–e179. 10.1016/S2214-109X(17)30429-1 29248367

[R40] Wakadha H, Chandir S, Were EV et al. 2013. The feasibility of using mobile-phone based SMS reminders and conditional cash transfers to improve timely immunization in rural Kenya. *Vaccine* 31: 987–93. 23246258 10.1016/j.vaccine.2012.11.093 PMC4603391

[R41] Warton EM, Parker MM. 2018. Oops I DID it again! Advanced Difference-in-Differences models in SAS. *Kaiser Permanente Division of Research* 2: 1–7.

[R42] Watterson JL, Walsh J, Madeka I. 2015. Using mHealth to improve usage of antenatal care, postnatal care, and immunization: a systematic review of the literature. *BioMed Research International* 2015: 153402. 10.1155/2015/153402 PMC4561933 26380263

[R43] WHO . 2011. mHealth: new horizons for health through mobile technologies: based on the findings of the second global survey on eHealth. *Global Observatory for eHealth Series* 3: 112.

[R44] WHO . 2021. Summary of interventions and evaluations by type of event. Scoping review of interventions to maintain essential services for maternal, newborn, child and adolescent health and older people during disruptive events. Geneva: WHO. Licence: CC BY-NC-SA 3.0 IGO. https://apps.who.int/iris/handle/10665/347601, accessed 12 March 2023.

[R45] WHO, UNICEF, UNFPA, World Bank Group, and the United Nations Population Division . 2019. Maternal mortality ratio (per 100,000 live births) estimates by WHO, UNICEF, UNFPA, World Bank Group, and the United Nations Population Division. WHO. https://www.who.int/data/gho/indicator-metadata-registry/imr-details/1911, accessed 11 March 2023.

