# Supplementary material for: The impact of digital interventions on health insurance coverage for reproductive, maternal, newborn and child health services utilization in Kakamega, Kenya: a cluster randomized controlled trial
Source: Health Policy Plan. 2024 Aug 24;39(10):1007–21. doi: 10.1093/heapol/czae079 (PMC11959180; doi:10.1093/heapol/czae079)
Supplement: czae079_Supp [file czae079_supp.zip › Supplementary Tables.docx]

**Table A1: Means and differences of RMNCH services utilization per survey wave**

| **Variables** | **Baseline** | | | | | **Midline** | | | | | **Endline** | | | | |
| --- | --- | --- | --- | --- | --- | --- | --- | --- | --- | --- | --- | --- | --- | --- | --- |
|  | **Control** | | **Treatment** | |  | **Control** | | **Treatment** | |  | **Control** | | **Treatment** | |  |
|  | **Mean** | **N1** | **Mean** | **N2** | **p-value** | **Mean** | **N1** | **Mean** | **N2** | **p-value** | **Mean** | **N1** | **Mean** | **N2** | **p-value** |
| **PANEL A. RMNCH utilization** | | | | | | | | | | | | | | | |
| Any ANC visits | 0.98 | 66 | 1.00 | 97 | 0.36 |  |  |  |  |  | 1.00 | 89 | 1.00 | 96 | . |
| Number of ANC visits | 2.55 | 66 | 2.68 | 97 | 0.28 |  |  |  |  |  | 2.92 | 89 | 2.93 | 96 | 0.97 |
| Tetanus toxoid injection at ANC | 0.59 | 66 | 0.63 | 97 | 0.75 |  |  |  |  |  | 0.81 | 89 | 0.71 | 96 | 0.07 |
| Iron supplements at ANC | 0.97 | 66 | 0.96 | 97 | 0.64 |  |  |  |  |  | 0.87 | 89 | 0.92 | 96 | 0.65 |
| Folic acid at ANC | 0.94 | 66 | 0.91 | 97 | 0.41 |  |  |  |  |  | 1.00 | 89 | 0.99 | 96 | 0.34 |
| Malaria prevention during pregnancy | 0.95 | 66 | 0.98 | 97 | 0.52 |  |  |  |  |  | 0.99 | 89 | 0.95 | 96 | 0.17 |
| Quality of ANC was good/very good | 0.89 | 65 | 0.85 | 97 | 0.49 |  |  |  |  |  | 0.90 | 89 | 0.92 | 96 | 0.72 |
| Delivery in health facility | 0.91 | 66 | 0.96 | 97 | 0.35 |  |  |  |  |  | 0.99 | 89 | 0.95 | 96 | 0.11 |
| Delivery with skilled birth attendant | 0.88 | 66 | 0.94 | 97 | 0.24 |  |  |  |  |  | 0.98 | 89 | 0.97 | 96 | 0.77 |
| Quality of delivery services was good/very good | 0.86 | 66 | 0.89 | 97 | 0.51 |  |  |  |  |  | 0.87 | 89 | 0.82 | 96 | 0.41 |
| Immediate PNC | 0.83 | 66 | 0.75 | 97 | 0.42 |  |  |  |  |  | 0.85 | 89 | 0.88 | 96 | 0.76 |
| PNC check-up after discharge | 0.15 | 66 | 0.16 | 97 | 0.83 |  |  |  |  |  | 0.20 | 89 | 0.14 | 96 | 0.37 |
| PNC received within 2 days | 0.06 | 66 | 0.02 | 97 | 0.31 |  |  |  |  |  | 0.11 | 89 | 0.04 | 96 | 0.25 |
| PNC for baby | 0.50 | 66 | 0.42 | 97 | 0.33 |  |  |  |  |  | 0.56 | 89 | 0.59 | 96 | 0.67 |
| **PANEL B. Family planning use** | | | | | | | | | | | | | | | |
| Any modern contraceptive | 0.45 | 137 | 0.50 | 143 | 0.49 | 0.68 | 130 | 0.67 | 145 | 0.90 | 0.58 | 117 | 0.66 | 131 | 0.32 |
| Short acting contraceptive | 0.17 | 137 | 0.28 | 143 | 0.05 | 0.16 | 130 | 0.22 | 145 | 0.32 | 0.15 | 117 | 0.23 | 131 | 0.39 |
| Long acting contraceptive | 0.28 | 137 | 0.22 | 143 | 0.22 | 0.52 | 130 | 0.47 | 145 | 0.57 | 0.44 | 117 | 0.44 | 131 | 0.95 |
| Unmet need for family planning | 0.32 | 100 | 0.27 | 112 | 0.37 | 0.16 | 106 | 0.18 | 114 | 0.69 |  |  |  |  |  |
| **PANEL C. Vaccination and bednet use** | | | | | | | | | | | | | | | |
| Child slept under bednet | 0.84 | 142 | 0.85 | 155 | 0.86 | 0.82 | 153 | 0.88 | 169 | 0.20 | 0.96 | 130 | 0.96 | 153 | 0.98 |
| Child fully vaccinated | 0.80 | 124 | 0.80 | 130 | 0.80 | 0.89 | 137 | 0.84 | 154 | 0.15 | 0.79 | 124 | 0.73 | 143 | 0.56 |
| Number of vaccinations (0-9) | 8.52 | 124 | 8.56 | 130 | 8.52 | 8.82 | 137 | 8.82 | 154 | 0.99 | 8.78 | 124 | 8.59 | 143 | 0.21 |

Notes: P-values are reported from Wald tests on the equality of means of Treatment and Comparison for each variable at each wave. Standard errors are clustered at the randomization pair (community)-level. In Panel A., most recent live births at midline and enline are merged and shown in the endline columns. In Panel B, data for unmet need was only available at baseline and midline.

**Table A2: General trends of RMNCH services utilization**

| **Variables** | **Baseline** | **Midline** | **Endline** | **Difference** | **Difference** |  |
| --- | --- | --- | --- | --- | --- | --- |
|  | **Mean** | **Mean** | **Mean** | **at midline** | **at endline** | **Observations** |
| **PANEL A. RMNCH healthcare utilization** | | | | | | |
| Any ANC visits | 0.994 |  | 1.000 |  | 0.006 | 348 |
| Number of ANC visits | 2.626 |  | 2.924 |  | 0.299** | 348 |
| Tetanus toxoid injection at ANC | 0.613 |  | 0.757 |  | 0.143* | 348 |
| Iron supplements at ANC | 0.963 |  | 0.892 |  | -0.071 | 348 |
| Folic acid at ANC | 0.920 |  | 0.995 |  | 0.074** | 348 |
| Malaria prevention during pregnancy | 0.969 |  | 0.968 |  | -0.002 | 348 |
| Quality of ANC was good/very good | 0.864 |  | 0.908 |  | 0.044 | 347 |
| Delivery in health facility | 0.939 |  | 0.968 |  | 0.029 | 348 |
| Delivery with skilled birth attendant | 0.914 |  | 0.973 |  | 0.059** | 348 |
| Quality of delivery services was good/very good | 0.877 |  | 0.843 |  | -0.034 | 348 |
| Immediate PNC | 0.785 |  | 0.865 |  | 0.080 | 348 |
| PNC check-up after discharge | 0.160 |  | 0.168 |  | 0.008 | 348 |
| PNC received within 2 days | 0.037 |  | 0.076 |  | 0.039 | 348 |
| PNC for baby | 0.454 |  | 0.578 |  | 0.124 | 348 |
| **PANEL B. Family planning use** |  |  |  |  |  |  |
| Any modern contraceptive | 0.475 | 0.679 | 0.621 | 0.204*** | 0.146** | 799 |
| Short acting contraceptive | 0.225 | 0.192 | 0.194 | -0.033 | -0.031 | 799 |
| Long acting contraceptive | 0.254 | 0.498 | 0.440 | 0.245*** | 0.186*** | 799 |
| Unmet need for family planning | 0.292 | 0.173 |  | -0.120** |  | 432 |
| **PANEL C. Vaccination and bednet use** | | | | | | |
| Child fully vaccinated | 0.799 | 0.866 | 0.757 | 0.067 | -0.043 | 812 |
| Number of vaccinations (0-9) | 8.543 | 8.818 | 8.678 | 0.275*** | 0.135 | 812 |
| Child slept under bednet | 0.842 | 0.854 | 0.961 | 0.012 | 0.119*** | 902 |

Notes: Significance tests are reported from Wald tests on the significance of the 'round' indicator. Standard errors are clustered at the pair level. In Panel A., most recent live births at midline and enline are merged and shown in the endline columns. In Panel B, data for unmet need was only available at baseline and midline.

**Table A3: Impact of *i*-PUSH on ANC and PNC outcomes (balanced panel sample)**

| **Outcome variable** | **Impact estimate** | **Baseline Treated** | **Baseline Control** | **Midline Treated** | **Midline Control** |
| --- | --- | --- | --- | --- | --- |
|  |  | **Mean** | **Mean** | **Mean** | **Mean** |
|  | **(1)** | **(2)** | **(3)** | **(4)** | **(5)** |
| Any ANC visits | -0.022 | 1.000 | 0.978 | 1.000 | 1.000 |
|  | (0.02) |  |  |  |  |
| At least 4 ANC visits | 0.051 | 0.740 | 0.689 | 0.880 | 0.778 |
|  | (0.10) |  |  |  |  |
| Tetanus toxoid injection at ANC | -0.170 | 0.620 | 0.600 | 0.760 | 0.911 |
|  | (0.13) |  |  |  |  |
| Iron supplements at ANC | 0.135 | 0.940 | 0.978 | 0.920 | 0.822 |
|  | (0.18) |  |  |  |  |
| Folic acid at ANC | 0.056* | 0.900 | 0.956 | 1.000 | 1.000 |
|  | (0.03) |  |  |  |  |
| Malaria prevention during pregnancy | -0.062 | 0.980 | 0.978 | 0.940 | 1.000 |
|  | (0.06) |  |  |  |  |
| Quality of ANC was good/very good | 0.167* | 0.820 | 0.909 | 0.920 | 0.844 |
|  | (0.08) |  |  |  |  |
| Delivery in health facility | -0.109 | 0.920 | 0.889 | 0.900 | 0.978 |
|  | (0.06) |  |  |  |  |
| Delivery with skilled birth attendant | -0.027 | 0.900 | 0.889 | 0.940 | 0.956 |
|  | (0.06) |  |  |  |  |
| Quality of delivery services was good/very good | -0.102 | 0.900 | 0.844 | 0.820 | 0.867 |
|  | (0.11) |  |  |  |  |
| Immediate PNC | 0.058 | 0.760 | 0.844 | 0.840 | 0.867 |
|  | (0.13) |  |  |  |  |
| PNC check-up after discharge | 0.004 | 0.160 | 0.133 | 0.120 | 0.089 |
|  | (0.09) |  |  |  |  |
| PNC received within 2 days | 0.002 | 0.040 | 0.067 | 0.020 | 0.044 |
|  | (0.03) |  |  |  |  |
| PNC for baby | 0.135 | 0.360 | 0.444 | 0.540 | 0.489 |
|  | (0.15) |  |  |  |  |
| **N** | **190** | **50** | **45** | **50** | **45** |

Standard errors in parentheses are clustered at the pair level. **p* < 0.1; ***p* < 0.05; ****p* < 0.01. Estimations use difference in difference modelling and include the following covariates: woman's age, women's literacy, household wealth, and pair fixed effects.

**Table A4: Impact of *i*-PUSH on family planning (balanced panel sample)**

| **Outcome variable** | **No. of Obs.** | **Control Mean,** | **Impact:** | **Impact** | **Impact Diff** |
| --- | --- | --- | --- | --- | --- |
|  |  | **Baseline** | **Midline** | **Endline** | **(EL vs ML)** |
|  |  | **(1)** | **(2)** | **(3)** | **(4)=(3)-(2)** |
| Any modern contraceptive | 573 | 0.475 | 0.057 | 0.074 | 0.017 |
|  |  |  | (0.09) | (0.10) | (0.06) |
|  |  |  |  |  |  |
| Short acting contraceptive | 573 | 0.192 | -0.086 | -0.045 | 0.041 |
|  |  |  | (0.08) | (0.10) | (0.08) |
|  |  |  |  |  |  |
| Long acting contraceptive | 573 | 0.283 | 0.154** | 0.140 | -0.014 |
|  |  |  | (0.07) | (0.09) | (0.08) |
|  |  |  |  |  |  |
| Unmet need for family planning | 322 | 0.289 | 0.059 |  |  |
|  |  |  | (0.11) |  |  |
|  |  |  |  |  |  |

Standard errors in parentheses are clustered at the pair level. **p* < 0.1; ***p* < 0.05; ****p* < 0.01. Estimations use difference in difference modelling and include the following covariates: woman's age, women's literacy, household wealth, and pair fixed effects. Data for unmet need was only available at baseline and midline.

**Table A5: Impact of *i*-PUSH on bednet use and vaccination uptake among children (balanced panel sample)**

| **Outcome variable** | **No. of Obs.** | **Control Mean,** | **Impact:** | **Impact** | **Impact Diff** |
| --- | --- | --- | --- | --- | --- |
|  |  | **Baseline** | **Midline** | **Endline** | **(EL vs ML)** |
|  |  | **(1)** | **(2)** | **(3)** | **(4)=(3)-(2)** |
| Child fully vaccinated | 396 | 0.833 | -0.061 | 0.061 | 0.122 |
|  |  |  | (0.09) | (0.14) | (0.16) |
|  |  |  |  |  |  |
| Number of vaccinations (0-9) | 396 | 8.591 | -0.183 | -0.227 | -0.044 |
|  |  |  | (0.17) | (0.22) | (0.23) |
|  |  |  |  |  |  |
| Child slept under bednet | 495 | 0.867 | -0.013 | -0.060 | -0.048 |
|  |  |  | (0.07) | (0.05) | (0.05) |
|  |  |  |  |  |  |

Standard errors in parentheses are clustered at the pair level. **p* < 0.1; ***p* < 0.05; ****p* < 0.01. Estimations use difference in difference modelling and include the following covariates: child's age, child sex, household wealth, and pair fixed effects.

**Table A6: Impacts on ANC and PNC outcomes (ANCOVA)**

| Indicator | Control Mean Baseline | Impact endline |
| --- | --- | --- |
|  | (1) | (2) |
| Any ANC visits | 0.978 | 0.000 |
|  |  |  |
| *N* | 45 | 95 |
| At least 4 ANC visits | 0.689 | 0.084 |
|  |  | (0.083) |
| *N* | 45 | 95 |
| Tetanus toxoid injection at ANC | 0.600 | -0.205*** |
|  |  | (0.039) |
| *N* | 45 | 95 |
| Iron supplements at ANC | 0.978 | 0.070 |
|  |  | (0.202) |
| *N* | 45 | 95 |
| Folic acid at ANC | 0.956 | 0.000 |
|  |  |  |
| *N* | 45 | 95 |
| Malaria prevention during ANC | 0.978 | -0.030 |
|  |  | (0.030) |
| *N* | 45 | 95 |
| Quality of ANC was good/very good | 0.909 | 0.086 |
|  |  | (0.057) |
| *N* | 44 | 94 |
| Delivery in health facility | 0.889 | -0.101** |
|  |  | (0.033) |
| *N* | 45 | 95 |
| Delivery with skilled birth attendant | 0.889 | -0.003 |
|  |  | (0.036) |
| *N* | 45 | 95 |
| Quality of delivery services was good/very good | 0.844 | -0.077 |
|  |  | (0.089) |
| *N* | 45 | 95 |
| Immediate PNC | 0.844 | 0.079 |
|  |  | (0.093) |
| *N* | 45 | 95 |
| PNC check-up after discharge | 0.133 | 0.069 |
|  |  | (0.067) |
| *N* | 45 | 95 |
| PNC received within 2 days | 0.067 | -0.005 |
|  |  | (0.040) |
| *N* | 45 | 95 |
| PNC for baby | 0.444 | 0.067 |
|  |  | (0.097) |
| *N* | 45 | 95 |

Standard errors in parentheses are clustered at the pair level. **p* < 0.1; ***p* < 0.05; ****p* < 0.01. Estimations use ANCOVA modelling and include the following covariates: baseline value of the outcome, woman's age, women's literacy, household wealth, and pair fixed-effects.

**Table A7: Impacts on family planning (ANCOVA)**

| **Indicator** | **No. of Obs.** | **Control Mean Baseline** | **Impact midline** | **Impact endline** | **Impact diff (EL vs ML)** |
| --- | --- | --- | --- | --- | --- |
|  |  | (1) | (2) | (3) | (4)=(3)-(2) |
| Any modern contraceptive | 382 | 0.475 | 0.085 | 0.016 | -0.069 |
|  |  |  | (0.068) | (0.064) | (0.097) |
|  |  |  |  |  |  |
| Short acting contraceptive | 382 | 0.192 | -0.002 | 0.041 | 0.043 |
|  |  |  | (0.080) | (0.084) | (0.136) |
|  |  |  |  |  |  |
| Long acting contraceptive | 382 | 0.283 | 0.112 | -0.014 | -0.126 |
|  |  |  | (0.080) | (0.083) | (0.134) |
|  |  |  |  |  |  |
| Unmet need for family planning | 166 | 0.289 | 0.021 |  |  |
|  |  |  | (0.080) |  |  |

Standard errors in parentheses are clustered at the pair level. **p* < 0.1; ***p* < 0.05; ****p* < 0.01. Estimations use ANCOVA modelling and include the following covariates: baseline value of the outcome, woman's age, women's literacy, household wealth, and pair fixed-effects. Data for unmet need was only available at baseline and midline.

**Table A8: Impacts on Vaccination coverage and bednet use among children (ANCOVA)**

| **Indicator** | **No. of Obs.** | **Control Mean Baseline** | **Impact midline** | **Impact endline** | **Impact diff (EL vs ML)** |
| --- | --- | --- | --- | --- | --- |
|  |  | (1) | (2) | (3) | (4)=(3)-(2) |
| Child fully vaccinated | 264 | 0.833 | -0.073 | 0.120 | 0.193 |
|  |  |  | (0.051) | (0.159) | (0.189) |
|  |  |  |  |  |  |
| Number of vaccinations (0-9) | 264 | 8.591 | -0.065 | -0.046 | 0.019 |
|  |  |  | (0.077) | (0.233) | (0.278) |
|  |  |  |  |  |  |
| Child slept under bednet | 330 | 0.867 | 0.029 | -0.048 | -0.076 |
|  |  |  | (0.055) | (0.049) | (0.098) |
|  |  |  |  |  |  |

Standard errors in parentheses are clustered at the pair level. **p* < 0.1; ***p* < 0.05; ****p* < 0.01. Estimations use ANCOVA modelling and include the following covariates: baseline value of the outcome, woman's age, women's literacy, household wealth, and pair fixed-effects.
